# Modified minimally invasive technique for decompression and reduction of thoracolumbar burst fracture with neurological symptoms: Technical Note

**DOI:** 10.1186/s13018-021-02783-x

**Published:** 2021-10-18

**Authors:** Xu Li, Zhiyuan Guan, Xiao Chen, Buzhou Chen, Lei Kong, Jintao Han, Wenzhi Zhang

**Affiliations:** 1 Spine Center, Department of Orthopedics, The First Affiliated Hospital of USTC, Division of Life Sciences and Medicine, University of Science and Technology of China, Hefei, 230001 Anhui People’s Republic of China; 2 Department of Orthopedics, The Shanghai Tenth People’s Hospital of Tongji University, Shanghai, People’s Republic of China; 3 Peking University Third Hospital Intervention and Vascular Surgery N0, 38 Xueyuan Road, Beijing, 100083 People’s Republic of China

**Keywords:** Thoracolumbar fracture, Neurological deficits, Percutaneous pedicle screw, Decompression under tube, Microscope

## Abstract

**Purpose:**

There are few reports about minimally invasive decompression and fixation for patients with thoracolumbar fracture and neurological symptoms. The previously reported method requires complete laminectomy, and removal of the medial part of the pedicle to expose the spinal canal for reduction. Thus, some approach-related damage to the bony structure and soft tissue still occurs. This study was performed to describe a modified minimally invasive tube technique for decompression and reduction of thoracolumbar fracture with neurological symptoms. This modified technique preserves most of the posterior structures of the spine as well as the muscle.

**Methods:**

Percutaneous pedicle screws were placed on the vertebrae superior and inferior to the fracture and at the fracture segment on the side with less severe symptoms. After retraction, the tube for decompression was placed on the facet joint where the decompression was needed. Under microscopic vision, part of the lamina and ligamentum flavum were removed to expose the spinal canal, and an L-shaped probe was used to reduce the bone fragment.

**Results:**

The modified method was successfully used in eight patients. Complete decompression was achieved and the bone fragment was safely reduced through the tube under microscopy in all cases. Fluoroscopy confirmed that the positioning of the percutaneous pedicle screw was good and the bone fragment was reduced. The neurological status was improved in all patients at last follow up.

**Conclusion:**

The modified method of minimally invasive decompression and fusion is effective in treating thoracolumbar fractures with neurological symptoms and preserves most of the ligaments and bone structure.

## Introduction

Surgery is the appropriate intervention for patients with thoracolumbar fracture with neurological symptoms[1–3]. The traditional open posterior approach is effective for such patients and is reportedly more effective in decompression than the anterior approach[4, 5]. However, the traditional incision for this approach is long and may cause approach-related complications. Minimally invasive procedures have been introduced to reduce the approach-related complications of spine surgery[6–8]; they have also been used for fixation and decompression in the treatment of patients with degenerative disease with neurological symptoms. Thus, the minimally invasive approach may also be suitable for thoracolumbar fracture with neurological symptoms.

Minimally invasive posterior decompression combined with percutaneous pedicle screw fixation was recently reported for the treatment of thoracolumbar fractures with neurological deficits[9]. This operation has outcomes similar to those of open reduction and fixation, with the advantages of less blood loss, less soft tissue damage, and faster recovery. However, this minimally invasive surgery still requires a 4-cm-long midline incision, complete laminectomy, and removal of the medial part of the pedicle to expose the spinal canal for reduction. Thus, there is still some approach-related damage to the bony structure and soft tissue.

The present report describes a modified minimally invasive way to complete decompression and reduction with less damage to the bony structure. In this proposed method, the retractor tube is placed on the facet joint on the side with more severe symptoms at the segment that needs to be decompressed.

## Methods

**Fig. 1:**
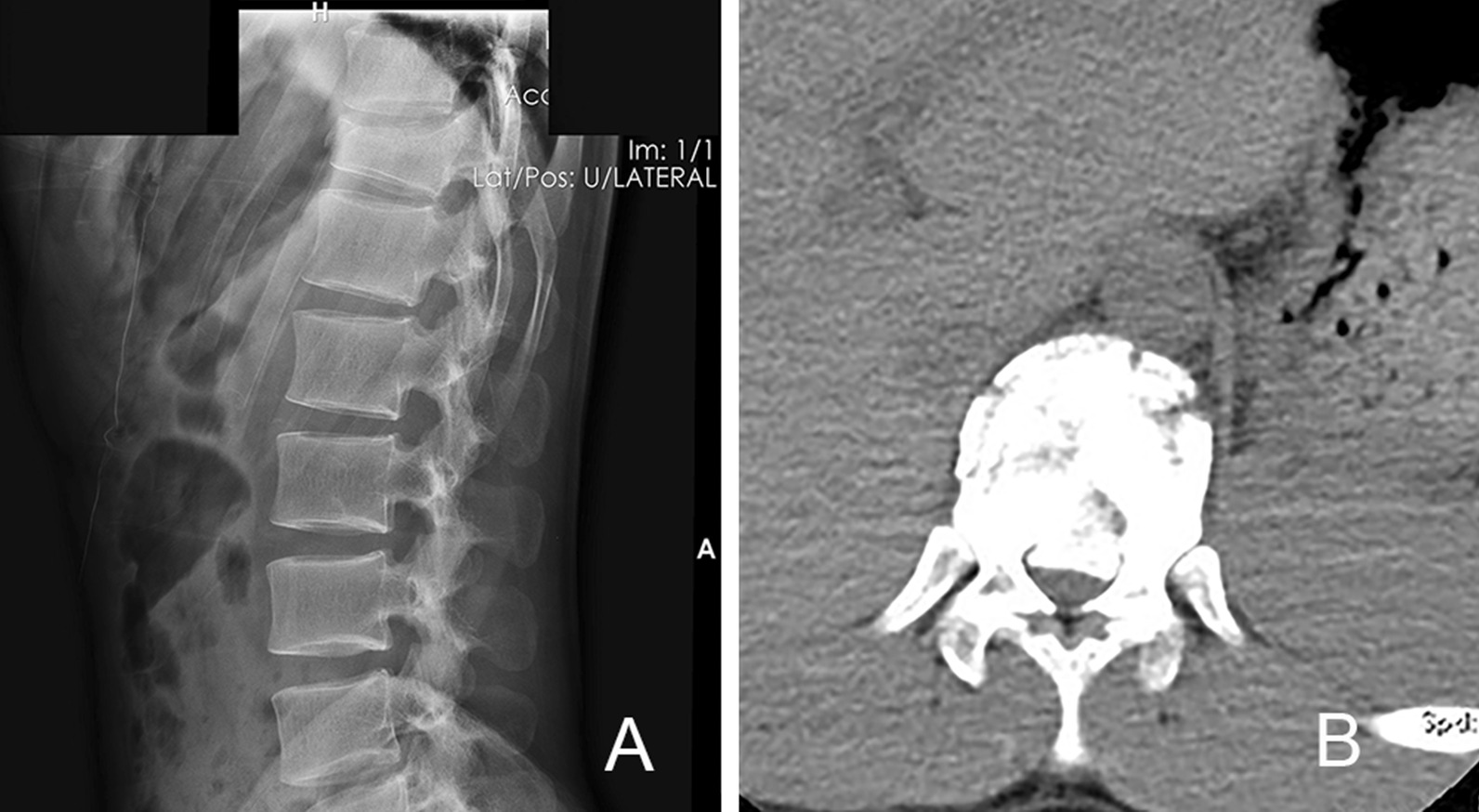
Radiograph of a 30-year-old patient who sustained a T11 burst fracture after a fall (**A**). Computed tomography showed that a large bone fragment had herniated into the spinal canal (**B**)

**Fig. 2:**
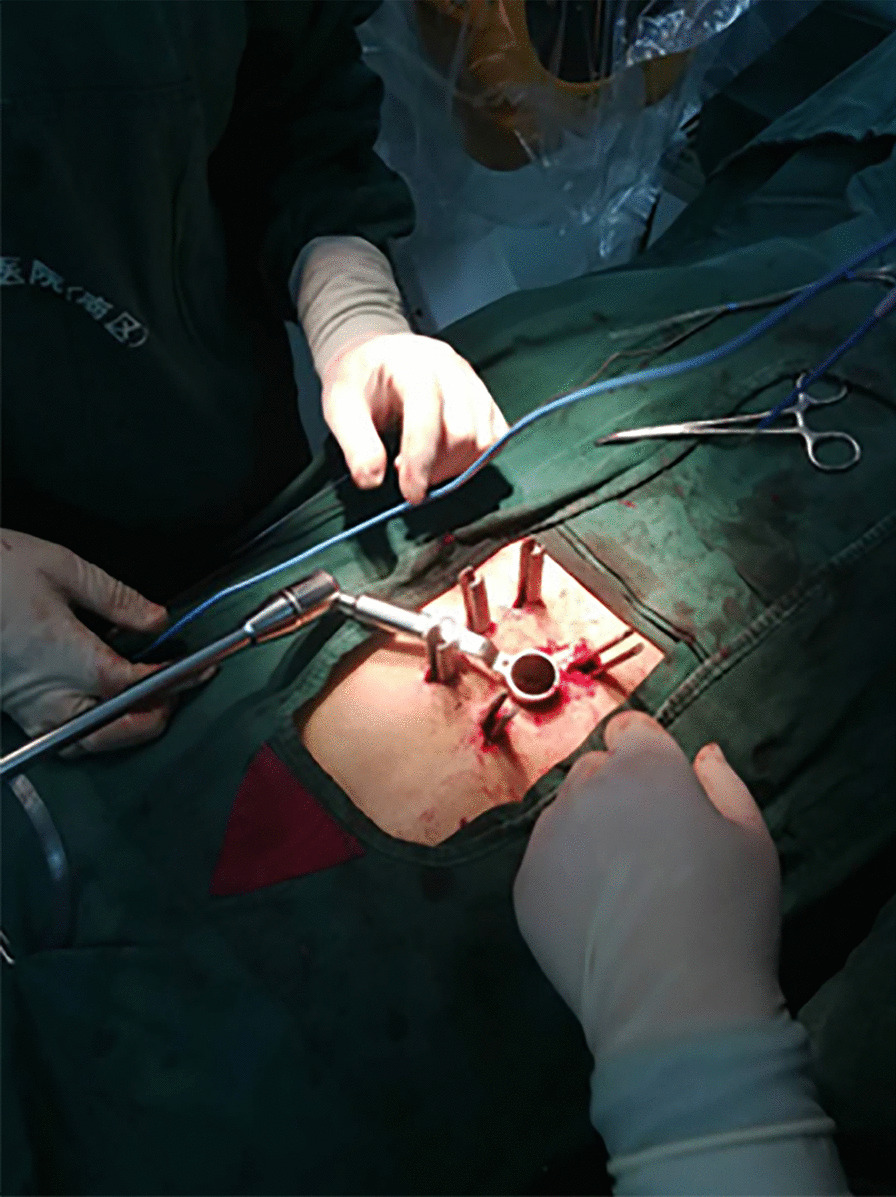
After placement of percutaneous pedicle screws at the pedicles and reduction by retraction on the screws, the rod on the decompression side was removed and a tube was placed on the facet joint

**Fig. 3:**
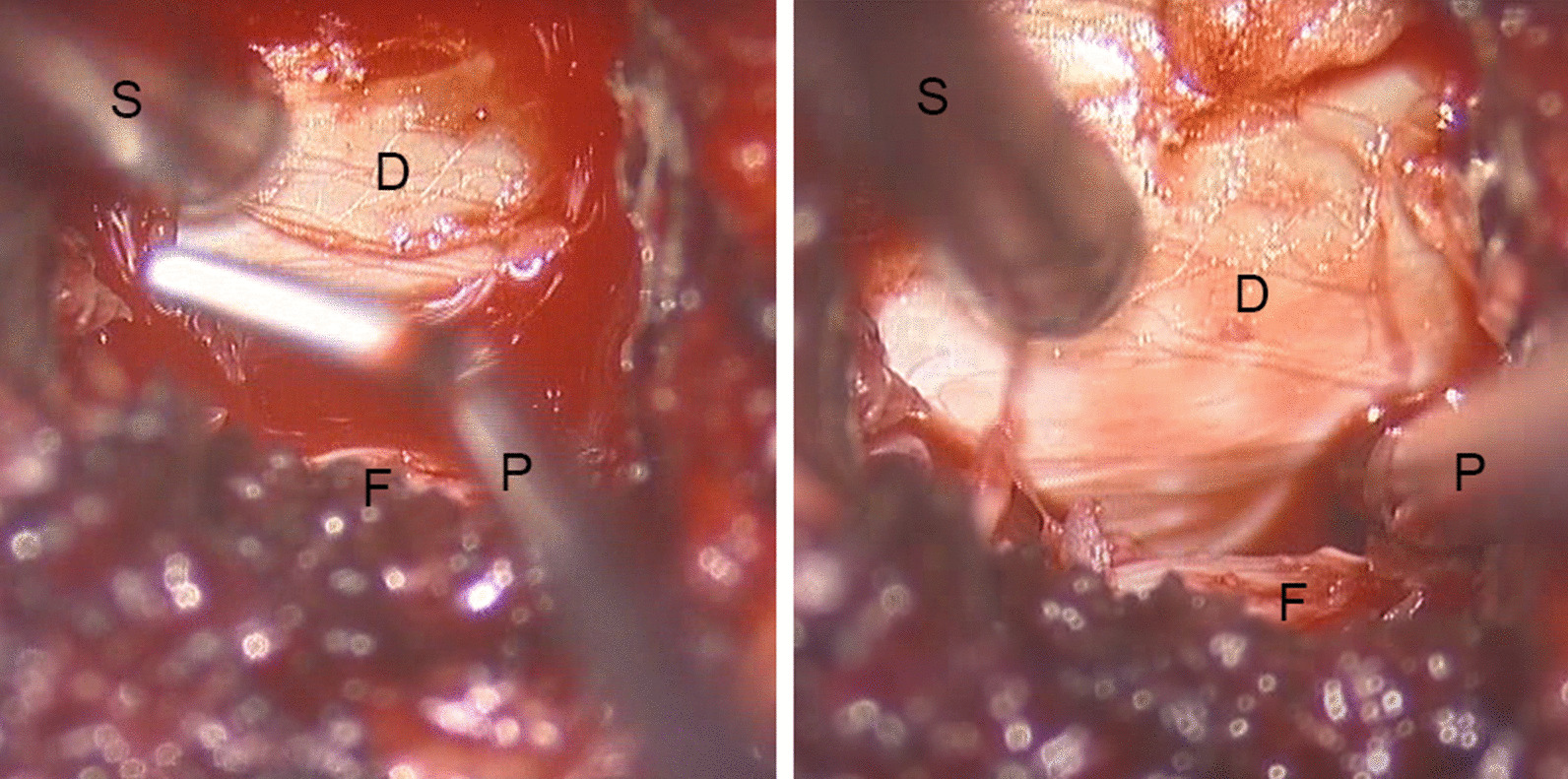
Image taken by a microscope. A probe (P) was used to reduce the bone fragment through the gap between the dura (D) and the pedicle, which could be detected along the facet joint (F). The suction tube (S) can also be seen in the picture

A modified method was applied in the treatment of thoracolumbar fracture with neurological symptoms. A radiographic examination (Fig. 1) and computed tomography scan of the fracture region were performed preoperatively. The projections of the pedicles were determined and marked on the skin before the start of the operation. Percutaneous pedicle screws (Fule Co. Beijing) were placed on the vertebrae superior and inferior to the fracture as well as at the fracture segment on the side with less severe symptoms. Rods were placed on both sides, and retraction was performed to reduce the fracture. The rod on the decompression side was then removed, and a 2.4-cm incision was made through the projection at the fracture segment. The incision was dilated by canals, and a tube (Medtronic. Inc) of the appropriate length was placed on the facet joint (Fig. 2). The microscope was adjusted and used to perform the decompression through the tube. The facet joint, lamina, and ligaments were carefully identified under microscopic vision. Part of the lamina and ligamentum flavum were then removed to expose the spinal canal. The facet joint was very carefully preserved. When the dura could be seen, an L-shaped probe was placed along the inner wall of the pedicle into the anterior part of the spinal canal (Fig. 3). The probe was used to detect the bone fragment and push it back into the vertebra. When necessary, a hammer was used to carefully tap the tail of the L-shaped probe to push the bone fragment back. The position of the bone fragment was then checked by fluoroscopy, and the reduction procedure was repeated if necessary. The rod was then replaced and fixed under distraction. The rest of the operation was performed in the same manner as the conventional technique.

## Results

**Fig. 4:**
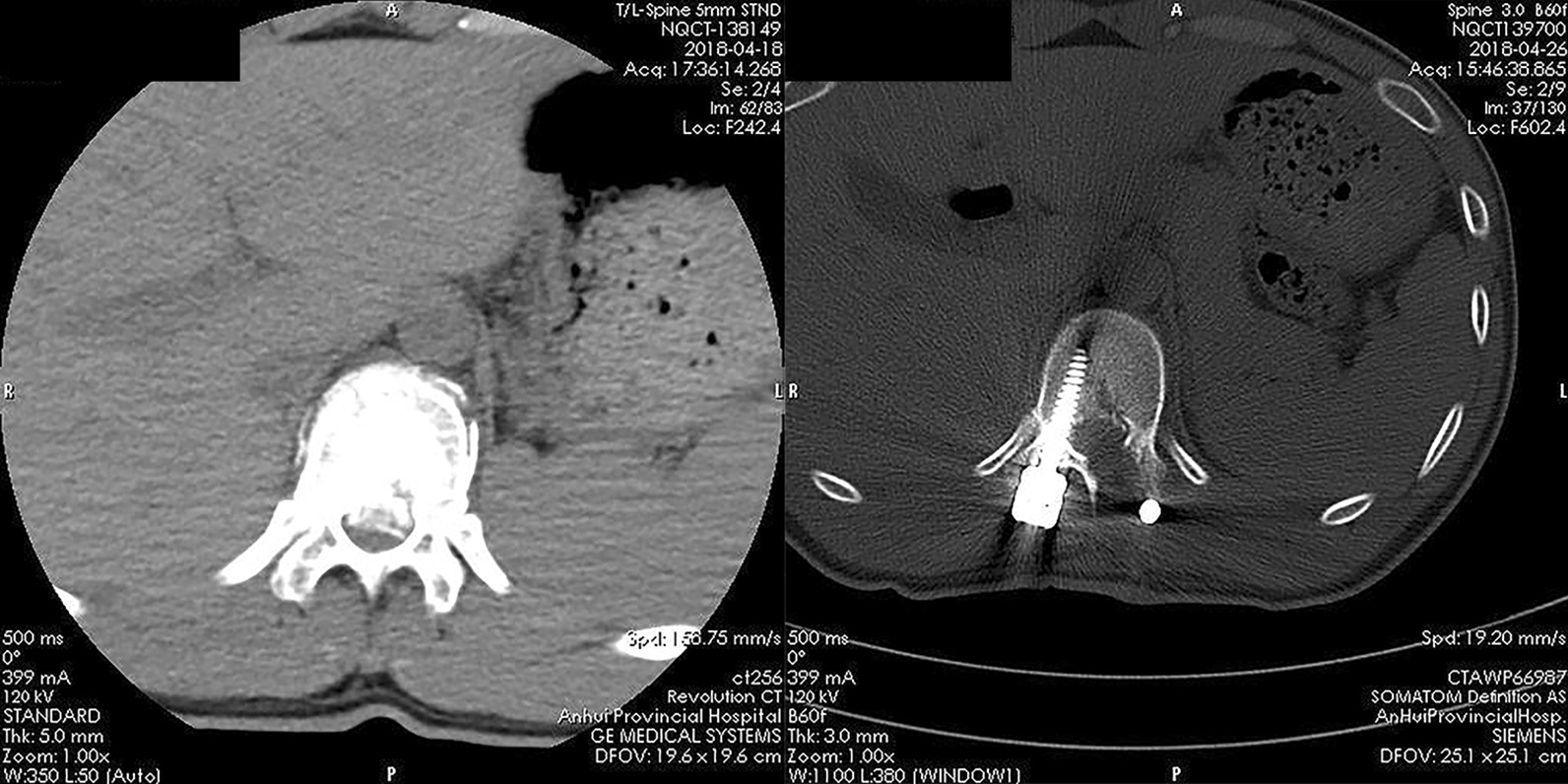
Computed tomography demonstrated that the bone fragment that had herniated into the spinal canal (**A**) was successfully reduced (**B**)

**Fig. 5:**
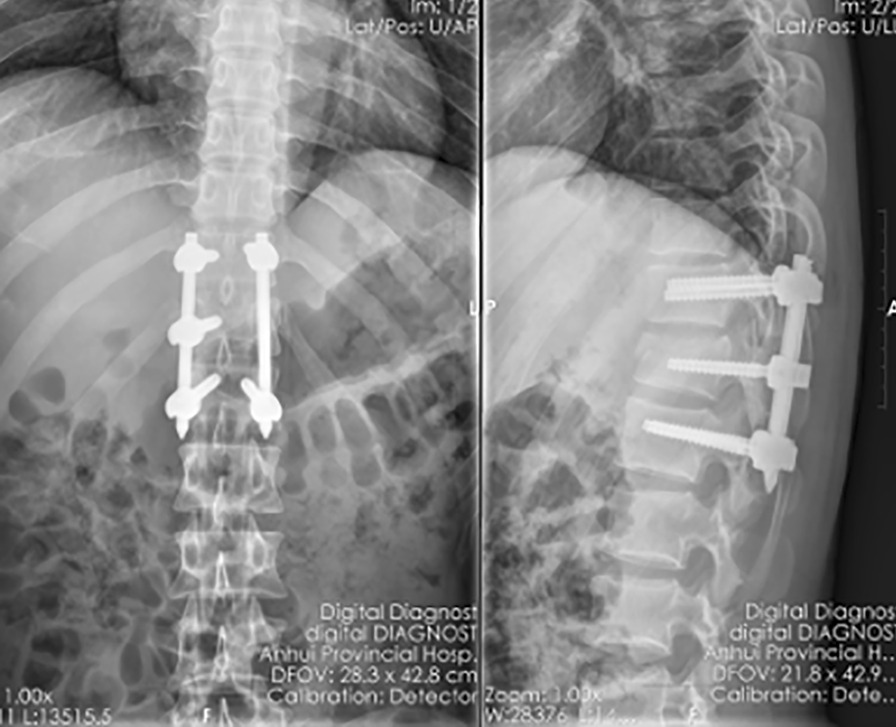
Postoperative radiograph. The fracture was reduced and the position of the screw was good

From 2017 to 2020, the modified technique was used to treat eight consecutive patients (3 males and 5 females), aged of 47(31–58) years, with thoracolumbar fracture with neurological symptoms and a bone fragment that had invaded the spinal canal and needed to be reduced(Table 1). The bone fragment was safely reduced through the tube under microscopic vision (Figs. 4, 5). The average operation time was 105 min (range, 85–130) min. All patients were able to get out of bed and sit on a wheelchair on the second day postoperatively. Two of them were able to walk with a brace. The lamina, facet joint, posterior ligament complex, and medial part of the pedicle were successfully preserved. The patients were followed up for 6–15 months. The neurological status was improved in all patients at last follow up. Bone healing of fracture was confirmed in every patient by X-ray. There was no deterioration and no patients developed complications.

**Table 1:** Patients' data

ID	Fracture level	Fixed level	TLICS score	AO type	ASIA pre-OP	ASIA post-OP	ASIA last-FU
1	L3	L2-4	5	A3	D	E	E
2	T11	T10-12	5	A3	C	C	D
3	L1	T12-L2	5	A3	D	D	E
4	L1	T11-L3	7	A3	C	C	D
5	L1	T12-L2	5	A4	D	D	E
6	L4	L3-5	5	A3	D	D	E
7	L1	T12-L2	5	A4	D	D	E
8	L3	L2-5	7	A4	C	C	E

## Discussion

In this study, a modified minimally invasive decompression and fixation method was used to treat eight patients with thoracolumbar fracture with neurological symptoms. The bone fragment was well reduced, and the neurological symptoms were improved postoperatively.

Minimally invasive procedures are performed to reduce the amount of approach-related damage. In the previously reported minimally invasive method for posterior decompression combined with percutaneous pedicle screw fixation for thoracolumbar fractures with neurological deficits, a 4-cm midline incision was made to place the tube, and the space available for the tube and the operative procedure was rather small because the spinous process and ligament were in the middle and the rods were on the outside. Furthermore, the muscle was stripped, and the lamina and part of the pedicle had to be removed to expose the spinal canal. In the herein-described modified method, the tube that was intended to conduct the decompression was placed on the facet joint, a 2.5-cm skin incision was made, and the muscle was separated rather than cut. Furthermore, only part of the lamina and ligamentum flavum were removed to expose the spinal canal, and the posterior structure of the spine was well preserved. This approach avoids the spinous process, and nothing hinders the tube. The tube can then be adjusted in any direction to obtain better exposure of the spine.

Reduction of the bone fragment responsible for the neurological symptoms is one of the most important goals of the operation. The bone fragments in most of the patients were located at the center or immediately lateral to the center of the spinal canal. The decompression approach was performed on the side with the more severe symptoms and the larger protrusion of the bone fragment. The bone fragment was partially reduced by retraction with screws. When the spinal canal was exposed, a microscope was used to enable more precise maneuvers and avoid damage to the dura. The L-shaped probe was placed along the inner wall of the pedicle into the gap between the wall and the dura. The length of the head of the L-shaped probe was 15 mm; because the mean length of the posterior vertebral part of the spinal canal is 26.3 ± 2.6 mm[10], the L-shaped probe could reach most of the bone fragment to push it back into the vertebrae. Because the diameter of the probe was only 1.5 to 2.0 mm, it did not cause further damage to the spinal cord. Neuromonitoring was suggested for patients with a bone fragment in the spinal canal at L1 and the thoracic segment.

**Fig. 6:**
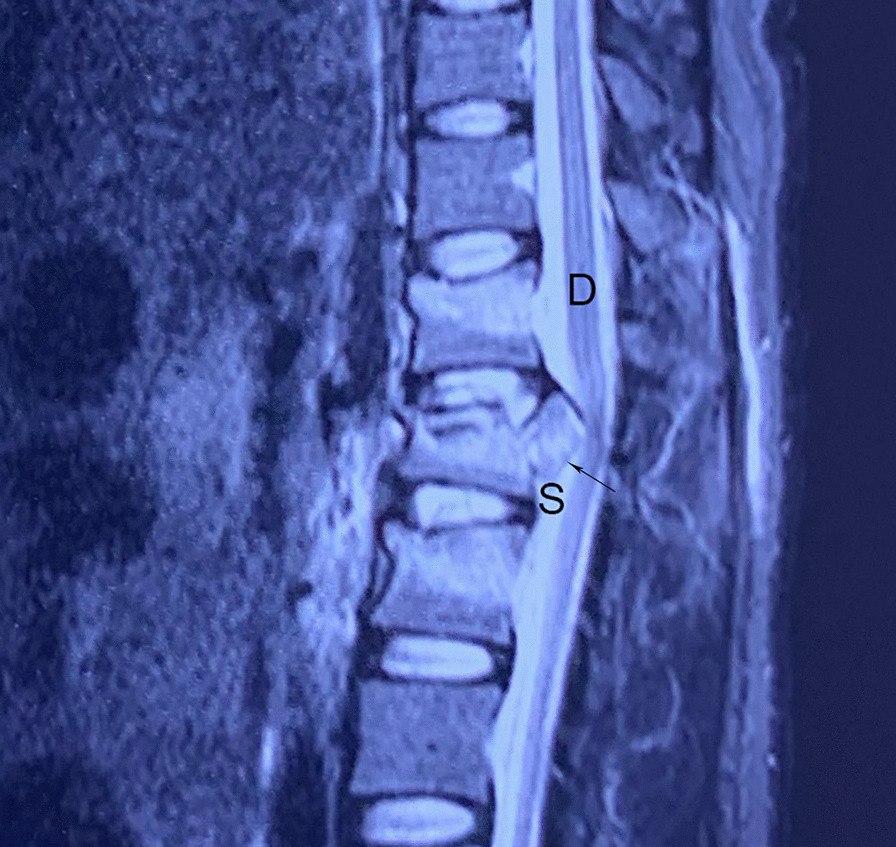
Magnetic resonance image of thoracic fracture. The bone fragment compressed the spinal cord (D). The subarachnoid space (S) can be seen between the slope (arrow) of the fragment and the spinal cord

In the thoracic spinal canal, wider exposure allowing for a more convergent path to the bone fragment appears to safely minimize the force exerted on the cord. The goal of the proposed method is exposure of the space between the dural sac and the inner wall of the pedicle. Based on the thoracic anatomy, some of the lamina and part of the facet joints were removed to expose the space. The probe was placed along the inner wall of the pedicle; the probe diameter was 2 mm, and it therefore just compressed the fluid in the subarachnoid space around the spinal cord when placed through the space. In most situations, the bone fragment was partially reduced by ligamentotaxis. If the bone fragment could not be reduced, cerebrospinal fluid was still present in the subarachnoid space between the slope of the bone fragment and the spinal cord (Fig. 6). The probe was placed on the slope to push the bone fragment back into the vertebra. The vertebral was partially reduced by the screws, and it was not difficult to push the bone fragment back. Throughout the whole process, the probe only disturbed the subarachnoid space; the spinal cord remained undisturbed.

The goal of surgery is to restore the stability and alignment of the spine and provide the possibility of early mobilization[7, 11, 12]. The proposed technique described in the present report involves the use of short-segment percutaneous fixation. The success rate of short-segment fixation is reportedly similar to that of long-segment fixation of burst fractures. Short-segment fixation interferes with fewer spinal segments and preserves more mobility of the spine than long-segment fusion[13]. Furthermore, the placement of a screw in the injured vertebra provides more stability in short-segment fixation[14–16]. Patients with thoracolumbar fracture may also have multiple comorbidities such as foot fractures, lower limb fractures, or neurological damage. Early fixation of the spine benefits the recovery of the whole body, making it easier to manage other injuries. In the present study, all patients were able to get out of bed and sit on a wheelchair on the second day postoperatively. Two of them were able to walk with a brace.

The posterior ligamentous complex[17, 18] is composed of the interspinous ligament, supraspinous ligament, ligamentum flavum, and facet joint capsules. This is an important structure that contributes to the stability of the spine by restricting the movement of the posterior spine column against translation, rotation, flexion, and distraction[19–21]. This structure is also well known for its poor ability to heal[2]. Because the tube and screws are placed through the muscle in the present modified method, most of the posterior ligamentous complex is kept intact, and the muscle is protected from extensive damage. Thus, the posterior stability of the spine is preserved as much as possible. The internal fixation device was removed 15 months after the operation. Because the segment was not fused in the posterior column, the flexibility of the segment was preserved as much as possible. The stability of the posterior column of the spine was not compromised by the surgery. When the fracture was healed, the anterior and middle column stability was reestablished.

The proposed method still has some limitations. Only small dorsal dural lesion can be repaired through the tube. It cannot be used when the bone fragment has been displaced too far from its original position. The exposure to the spinal canal is fairly small, and the probe can only reach the posterior part of the vertebra at 1 cm superior or inferior to the pedicle level. If the bone fragment migrates outside this area, it is difficult to reduce using the proposed technique. Additionally, rotated bone fragments are not suited for this technique. Because most of the lamina and facet joints are preserved, the spinal canal is not expanded. In such a narrow space, reduction of the rotated bone fragment may cause disturbance to the spinal cord.

## Conclusion

The modified method of minimally invasive decompression and fusion seems to be effective for the treatment of thoracolumbar diseases, enabling preservation of most of the ligaments and bone structure.

## Data Availability

All data generated or analysed during this study are included in this published article.

## References

[1] Vaccaro AR, Kim DH, Brodke DS (2004). Diagnosis and management of thoracolumbar spine fractures. Instr Course Lect.

[2] Vaccaro AR, Lehman RA, Hurlbert RJ (2005). A new classification of thoracolumbar injuries: the importance of injury morphology, the integrity of the posterior ligamentous complex, and neurologic status. Spine.

[3] Radcliff KE, Kepler CK, Delasotta LA, et al. Current management review of thoracolumbar cord syndromes. Spine J 2011;**11**(9):884–92 doi: 10.1016/j.spinee.2011.07.022[published Online First: Epub Date]|. 10.1016/j.spinee.2011.07.022 21889419

[4] Jiang XZ, Tian W, Liu B, et al. Comparison of a paraspinal approach with a percutaneous approach in the treatment of thoracolumbar burst fractures with posterior ligamentous complex injury: a prospective randomized controlled trial. J Int Med Res 2012;**40**(4):1343–56 doi: 10.1177/147323001204000413[published Online First: Epub Date]|. 10.1177/147323001204000413 22971486

[5] Zhu Q, Shi F, Cai W, Bai J, Fan J, Yang H. Comparison of anterior versus posterior approach in the treatment of thoracolumbar fractures: a systematic review. Int Surg 2015;**100**(6):1124–33 doi: 10.9738/INTSURG-D-14-00135.1[published Online First: Epub Date]|. 10.9738/INTSURG-D-14-00135.1 PMC4587517 26414835

[6] Wood KB, Buttermann GR, Phukan R, et al. Operative compared with nonoperative treatment of a thoracolumbar burst fracture without neurological deficit: a prospective randomized study with follow-up at sixteen to twenty-two years. J Bone Joint Surg Am 2015;**97**(1):3–9 doi: 10.2106/JBJS.N.00226[published Online First: Epub Date]|. 10.2106/JBJS.N.00226 25568388

[7] Gong Y, Fu G, Li B, Li Y, Yang X. Comparison of the effects of minimally invasive percutaneous pedicle screws osteosynthesis and open surgery on repairing the pain, inflammation and recovery of thoracolumbar vertebra fracture. Exp Ther Med 2017;**14**(5):4091–96 doi: 10.3892/etm.2017.5036[published Online First: Epub Date]|. 10.3892/etm.2017.5036 PMC5647694 29067101

[8] Malham GM. Minimally invasive direct lateral corpectomy for the treatment of a thoracolumbar fracture. J Neurol Surg Part A, Central Eur Neurosurg 2015;**76**(3):240–3 doi: 10.1055/s-0034-1368094[published Online First: Epub Date]|. 10.1055/s-0034-1368094 24793062

[9] Zhang W, Li H, Zhou Y, et al. Minimally invasive posterior decompression combined with percutaneous pedicle screw fixation for the treatment of thoracolumbar fractures with neurological deficits: a prospective randomized study versus traditional open posterior surgery. Spine 2016;**41 Suppl 19**:B23-B29 doi: 10.1097/BRS.0000000000001814[published Online First: Epub Date]|. 10.1097/BRS.0000000000001814 27656782

[10] Vaccaro AR, Rizzolo SJ, Allardyce TJ, et al. Placement of pedicle screws in the thoracic spine. Part I: Morphometric analysis of the thoracic vertebrae. J Bone Joint Surg Am 1995;**77**(8):1193–9 10.2106/00004623-199508000-00008 7642664

[11] Gu Y, Zhang F, Jiang X, Jia L, McGuire R. Minimally invasive pedicle screw fixation combined with percutaneous vertebroplasty in the surgical treatment of thoracolumbar osteoporosis fracture. J Neurosurg Spine 2013;**18**(6):634–40 doi: 10.3171/2013.3.SPINE12827[published Online First: Epub Date]|. 10.3171/2013.3.SPINE12827 23560713

[12] Laghmouche N, Prost S, Farah K, Graillon T, Blondel B, Fuentes S. Minimally invasive treatment of thoracolumbar flexion-distraction fracture. Orthopaedics Traumatol Surg Res OTSR 2019;**105**(2):347–50 doi: 10.1016/j.otsr.2018.09.023[published Online First: Epub Date]|. 10.1016/j.otsr.2018.09.023 30792168

[13] Dai LY, Jiang LS, Jiang SD. Posterior short-segment fixation with or without fusion for thoracolumbar burst fractures. a five to seven-year prospective randomized study. J Bone Joint Surg Am 2009;**91**(5):1033–41 doi: 10.2106/JBJS.H.00510[published Online First: Epub Date]|. 10.2106/JBJS.H.00510 19411450

[14] Mahar A, Kim C, Wedemeyer M, et al. Short-segment fixation of lumbar burst fractures using pedicle fixation at the level of the fracture. Spine 2007;**32**(14):1503–7 doi: 10.1097/BRS.0b013e318067dd24[published Online First: Epub Date]|. 10.1097/BRS.0b013e318067dd24 17572619

[15] Wang H, Zhao Y, Mo Z, et al. Comparison of short-segment monoaxial and polyaxial pedicle screw fixation combined with intermediate screws in traumatic thoracolumbar fractures: a finite element study and clinical radiographic review. Clinics (Sao Paulo) 2017;**72**(10):609–17 doi: 10.6061/clinics/2017(10)04[published Online First: Epub Date]|. 10.6061/clinics/2017(10)04 PMC5666442 29160423

[16] Zhang C, Liu Y. Combined pedicle screw fixation at the fracture vertebrae versus conventional method for thoracolumbar fractures: A meta-analysis. Int J Surg 2018;**53**:38–47 doi: 10.1016/j.ijsu.2018.03.002[published Online First: Epub Date]|. 10.1016/j.ijsu.2018.03.002 29535015

[17] Hartmann F, Nusselt T, Mattyasovszky S, Maier G, Rommens PM, Gercek E. Misdiagnosis of thoracolumbar posterior ligamentous complex injuries and use of radiographic parameter correlations to improve detection accuracy. Asian Spine J 2019;**13**(1):29–34 doi: 10.31616/asj.2017.0333[published Online First: Epub Date]|. 10.31616/asj.2017.0333 PMC6365780 30326695

[18] Beausejour MH, Petit Y, Hagen J, Arnoux PJ, Thiong JM, Wagnac E. Contribution of injured posterior ligamentous complex and intervertebral disc on post-traumatic instability at the cervical spine. Comput Methods Biomech Biomed Engin 2020;**23**(12):832–43 doi: 10.1080/10255842.2020.1767776[published Online First: Epub Date]|. 10.1080/10255842.2020.1767776 32463324

[19] Rajasekaran S, Maheswaran A, Aiyer SN, Kanna R, Dumpa SR, Shetty AP. Prediction of posterior ligamentous complex injury in thoracolumbar fractures using non-MRI imaging techniques. Int Orthop 2016;**40**(6):1075–81 doi: 10.1007/s00264-016-3151-1[published Online First: Epub Date]|. 10.1007/s00264-016-3151-1 26983409

[20] Mi J, Sun XJ, Zhang K, Zhao CQ, Zhao J. Prediction of MRI findings including disc injury and posterior ligamentous complex injury in neurologically intact thoracolumbar burst fractures by the parameters of vertebral body damage on CT scan. Injury 2018;**49**(2):272-78 10.1016/j.injury.2017.12.011 29290375

[21] Wu CC, Jin HM, Yan YZ, et al. Biomechanical Role of the Thoracolumbar Ligaments of the Posterior Ligamentous Complex: A Finite Element Study. World neurosurgery 2018;**112**:e125-e33 doi: 10.1016/j.wneu.2017.12.171[published Online First: Epub Date]|. 10.1016/j.wneu.2017.12.171 29317367

